# Supplementary material for: Simulation of the NMR response in the pseudogap regime of the cuprates
Source: Nat Commun. 2017 Apr 7;8:14986. doi: 10.1038/ncomms14986 (PMC5385573; doi:10.1038/ncomms14986)
Supplement: Supplementary Information — Supplementary Figures, Supplementary Discussion, Supplementary Methods, Supplementary Results and Supplementary References [file ncomms14986-s1.pdf]

Supplemental material for: Simulation of the NMR response in the pseudogap regime of the cuprates

Xi Chen, J. P. F. LeBlanc, and Emanuel Gull
Department of Physics, University of Michigan, Ann Arbor, Michigan 48109, USA
(Dated: December 19, 2016)

I. SUPPLEMETARY DISCUSSION

This supplement presents additional information, tests, validations, and side-by-side comparisons for the manuscript. Section II A describes the formalism for obtaining generalized susceptibilities in more details. Section II B relates those susceptibilities to quantities measured in Nuclear Magnetic Resonance. Section II C presents the single-particle spectral functions that were used to compare the pseudogap to the NMR gap. Section III A shows comparison data for the relaxation time between our work and random phase approximation (RPA) data in a regime where the RPA is believed to be reliable. Finally, section III B shows side-by-side comparisons of the experimental data cited in the main text to our calculations.

II. SUPPLEMENTARY METHODS

A. Generalized Susceptibility

We define the one-particle and two-particle Green's functions in imaginary time, τ as

$$G_{\sigma_1\sigma_2}(k_1\tau_1, k_2\tau_2) = \langle T_\tau(c^\dagger_{k_1\sigma_1}(\tau_1)c_{k_2\sigma_2}(\tau_2)\rangle \tag{1}$$

$$G_{2,\sigma_1\sigma_2\sigma_3\sigma_4}(k_1\tau_1, ..., k_4\tau_4) = \langle T_\tau(c^\dagger_{k_1\sigma_1}(\tau_1)c_{k_2\sigma_2}(\tau_2)c^\dagger_{k_3\sigma_3}(\tau_3)c_{k_4\sigma_4}(\tau_4)\rangle. \tag{2}$$

The generalized susceptibility can be written in imaginary time, τ, in terms of the one- and two-particle Green's functions as[1]

$$\chi_{\sigma_1\sigma_2\sigma_3\sigma_4}(k_1\tau_1, k_2\tau_2, k_3\tau_3, k_4\tau_4) = G_{2,\sigma_1...\sigma_4}(k_1\tau_1, k_2\tau_2, k_3\tau_3, k_4\tau_4) - G_{\sigma_1\sigma_2}(k_1\tau_1, k_2\tau_2)G_{\sigma_3\sigma_4}(k_3\tau_3, k_4\tau_4).$$

The susceptibility can be represented in frequency space via a Fourier transform. In the particle-hole (ph) convention, it is defined as

$$\chi^{\omega\omega'\nu}_{ph\sigma\sigma'}(k, k', q) = \int_0^\beta \int_0^\beta \int_0^\beta d\tau_1 d\tau_2 d\tau_3 \chi_{\sigma\sigma\sigma'\sigma'}(k\tau_1, (k'+q)\tau_2, (k+q)\tau_3, k'0)e^{-i\omega\tau_1}e^{i(\omega+\nu)\tau_2}e^{-i(\omega'+\nu)\tau_3}$$

where ω and ω' are fermionic Matsubara frequencies, ν is a bosonic Matsubara frequency, σ and σ' are $\uparrow$ or $\downarrow$ spin labels and k, k' and q are initial, final and transfer momenta respectively. The spin susceptibility is the difference between the $\sigma\sigma' \equiv \uparrow\uparrow$ susceptibility and the $\uparrow\downarrow$ susceptibility,

$$\chi_m = \chi_{ph\uparrow\uparrow} - \chi_{ph\uparrow\downarrow}. \tag{3}$$

For the paramagnetic case, we define the bare susceptibility as

$$\chi^{\omega\omega'\nu}_{0ph}(k, k', q) = -\beta G_{\sigma\sigma}(k, i\omega)G_{\sigma\sigma}(q+k, i\nu+i\omega)\delta_{\omega\omega'}\delta_{kk'}, \tag{4}$$

where the spin index has been omitted on the left hand side due to spin degeneracy. The Bethe-Salpeter equation in the magnetic channel is[1]

$$\chi^{\omega\omega'\nu}_m(k, k', q) = \chi^{\omega\omega'\nu}_{0ph}(k, k', q) - \frac{1}{\beta^2}\chi^{\omega\omega''\nu}_{0ph}(k, k'', q)\Gamma^{\omega''\omega'''\nu}_m(k'', k''', q)\chi^{\omega'''\omega'\nu}_m(k''', k', q). \tag{5}$$

where repeated indices are summed over. This equation defines the vertex function Γ.

Within the DCA approximation[2], the single particle lattice selfenergy is coarse grained from its cluster counterpart, $\Sigma_\sigma(k,i\omega) = \Sigma_\sigma(K+\tilde{k},i\omega) \approx \Sigma_\sigma(K,i\omega)$, where K defines a cluster vector and $\tilde{k}$ a vector within a momentum 'patch'.

Similarly the bare lattice susceptibility

$$\overline{\chi}_{0ph}^{\omega\omega'\nu}(K,K',Q) = -\beta\frac{N_c}{N}\sum_{\tilde{k}} G_\sigma(K+\tilde{k},i\omega)G_\sigma(Q+K+\tilde{k},i\nu+i\omega)\delta_{\omega\omega'}\delta_{KK'}, \quad (6)$$

and, as described in detail in Fotso *et al.* [3], we perform an approximation to the vertex function, $\Gamma = \frac{\delta\Sigma}{\delta G}$:

$$\bar{\Gamma}_m^{\omega\omega'\nu}(K,K',Q) \to \Gamma_{cm}^{\omega\omega'\nu}(K,K',Q). \quad (7)$$

We then sum over lattice momenta within one patch of k-space to get coarse-grained equation (5) as

$$\overline{\chi}_m^{\omega\omega'\nu}(K,K',Q) = \overline{\chi}_{0ph}^{\omega\omega'\nu}(K,K',Q) - \frac{1}{\beta^2}\overline{\chi}_{0ph}^{\omega\omega''\nu}(K,K'',Q)\Gamma_{cm}^{\omega''\omega'''\nu}(K'',K''',Q)\overline{\chi}_m^{\omega'''\omega'\nu}(K''',K',Q). \quad (8)$$

Cluster quantities also follow the Bethe-salpeter equation,

$$\chi_m^{\omega\omega'\nu}(K,K',Q) = \chi_{0cph}^{\omega\omega'\nu}(K,K',Q) - \frac{1}{\beta^2}\chi_{0cph}^{\omega\omega''\nu}(K,K'',Q)\Gamma_{cm}^{\omega''\omega'''\nu}(K'',K''',Q)\chi_{cm}^{\omega'''\omega'\nu}(K''',K',Q). \quad (9)$$

Combining equations (8) and (9) to eliminate the cluster vertex, Γ_c, yields

$$\overline{\chi}^{-1} = \chi_c^{-1} - \chi_{0c}^{-1} + \overline{\chi}_0^{-1} \quad (10)$$

where $\overline{\chi}$, χ_c, χ_{0c} and $\overline{\chi}_0$ are all matrices in cluster momentum K and frequency ω. This procedure is described in detail in Ref. 2.

B. NMR related quantities: K^S, T_{2g} and T_1

The spin-lattice relaxation rate $1/T_1$ is related to the imaginary part of dynamical spin susceptibility on the real frequency axis as

$$\frac{1}{T_1T} \propto \lim_{\nu\to 0}\sum_q {}^{\alpha}F_{\parallel}(q)\frac{\chi_m''(q,\nu)}{\nu}, \quad (11)$$

where ${}^{\alpha}F_{\parallel}(q)$ differs for ^{63}Cu and ^{17}O, as defined in Ref. 4. For the two isotopes used in this paper,

$$\begin{aligned} {}^{63}F_{\parallel} &= A_\perp + 2B[\cos(q_x)+\cos(q_y)]^2 \\ {}^{17}F_{\parallel} &= 2C_{\parallel}^2[1+0.5[\cos(q_x)+\cos(q_y)]] \\ A_\perp &= 0.84B, C_{\parallel} = 0.91B. \end{aligned} \quad (12)$$

Obtaining $\chi''_m(q,\nu)/\nu$ from Matsubara frequency data requires analytical continuation. If temperature is low enough, we can consider the parametrization

$$\chi''_m(q,\nu) = \nu\chi_m(q,0)/\Gamma_q. \tag{13}$$

We can then write the spin structure factor, $S(q,\tau)$ at time $\tau=\beta/2$ as

$$S(q,\tau=\frac{\beta}{2}) = \int d\nu \frac{\chi_m(q,0)\nu}{\Gamma_q \sinh\frac{\nu}{2T}} = \frac{\chi_m(q,0)}{\Gamma_q}\int \frac{4T^2\lambda d\lambda}{\sinh\lambda} = T^2\pi^2\frac{\chi_m(q,0)}{\Gamma_q} \tag{14}$$

where $S(q,\tau) = \langle s^z_q(\tau)s^z_{-q}(0)\rangle$ and S^z_q is the real-to-k-space Fourier transform of $s^z_i = n_{i\uparrow} - n_{i\downarrow}$. To connect $S(q,\tau)$ with spin susceptibility,

$$\begin{aligned} S(q,\tau) = \langle s^z_q(\tau)s^z_{-q}(0)\rangle &= \frac{1}{N}\langle\sum_{R_iR_j} e^{-iq(R_i-R_j)} \times [n_{i\uparrow}(\tau)-n_{i\downarrow}(\tau)][n_{j\uparrow}(0)-n_{j\downarrow}(0)]\rangle \\ &= \frac{1}{N}\langle\sum_{R_iR_j} e^{-iq(R_i-R_j)} \times [c^\dagger_{i\uparrow}c_{i\uparrow}c^\dagger_{j\uparrow}c_{j\uparrow} - c^\dagger_{i\downarrow}c_{i\downarrow}c^\dagger_{j\uparrow}c_{j\uparrow} - c^\dagger_{i\uparrow}c_{i\uparrow}c^\dagger_{j\downarrow}c_{j\downarrow} + c^\dagger_{i\downarrow}c_{i\downarrow}c^\dagger_{j\downarrow}c_{j\downarrow}]\rangle \end{aligned} \tag{15}$$

where we have omitted τ in the equation. Note that creation/annihilation operator site i has imaginary time τ and site j $\tau=0$.

$$\frac{1}{\sqrt{N}}\sum_q c_q e^{iqR_i} = c_i,\ \frac{1}{\sqrt{N}}\sum_q c^\dagger_q e^{-iqR_i} = c^\dagger_i \tag{16}$$

Omitting spin indices for now, the terms in equation 15 become

$$\frac{1}{N^3}[\sum_{k_1k_2}(\sum_{R_i} e^{iR_i(-q+k_1-k_2)})c^\dagger_{k_1}c_{k_2}][\sum_{p_1p_2}(\sum_{R_j} e^{iR_j(q+p_1-p_2)})c^\dagger_{p_1}c_{p_2}] \quad = \frac{1}{N}\sum_{k_2} c^\dagger_{k_2+q}c_{k_2}\sum_{p_2} c^\dagger_{p_2-q}c_{p_2} \tag{17}$$

There are four terms like this, with spin ↑↑↑↑, ↓↓↑↑, ↑↑↓↓, ↓↓↓↓ separately. Given the symmetry of 2-particle Green's function, we find

$$\begin{aligned} S(q,\tau) = \frac{1}{N}\langle(2\sum_{kk'} c^\dagger_{k+q\uparrow}(\tau)c_{k\uparrow}(\tau)c^\dagger_{k'\uparrow}(0)c_{k'+q\uparrow}(0) \\ -2\sum_{kk'} c^\dagger_{k+q\uparrow}(\tau)c_{k\uparrow}(\tau)c^\dagger_{k'\downarrow}(0)c_{k'+q\downarrow}(0))\rangle \end{aligned} \tag{18}$$

where $k=k_2$, $k'=p_2-q$. This is the magnetic channel susceptibility

$$S(q,\tau) = \frac{1}{N}\sum_{kk'}\chi_m(k+q,\tau;k,\tau;k',0;k'+q,0) = \frac{2}{N}\sum_{kk'}\chi^{kk'q}_m(\tau,\tau,0). \tag{19}$$

Fourier transforming it to Matsubara frequency space, we obtain

$$S(q,\tau) = \frac{2}{N\beta^3}\sum_{kk',\omega\omega'\nu}\chi^{\omega\omega'\nu}_m(k,k',q)e^{-i\nu\tau} \tag{20}$$

Within DCA,

$$\sum_q {}^{\alpha}F_{\|}(q)S(q,\tau) = \frac{2}{N\beta^3}(\frac{N}{N_c})^3 \sum_{KK'Q} \sum_{\omega\omega'\nu} {}^{\alpha}F_{\|}(Q)\chi_m^{\omega\omega'\nu}(K,K',Q)e^{-i\nu\tau} = \frac{2}{\beta}\sum_{Q,\nu} {}^{\alpha}F_{\|}(Q)\chi_m(Q,i\nu)e^{-i\nu\tau} \tag{21}$$

And combining Eq. 13, Eq. 14 and Eq. 21 yields

$$\frac{1}{T_1} = \frac{\sum_q {}^{\alpha}F_{\|}(q)S(q,\tau=\beta/2)}{\pi^2 T} = \frac{2}{\beta\pi^2 T}\sum_{Q,\nu} {}^{\alpha}F_{\|}(Q)\chi_m(Q,i\nu)e^{-i\nu\beta/2} \tag{22}$$

$$= \frac{2}{\pi^2}\sum_{Q,n} {}^{\alpha}F_{\|}(Q)\chi_m(Q,i\nu_n)(-1)^n. \tag{23}$$

According to the Mila-Rice-Shastry model for HF coupling with itinerant Cu^{2+} holes in high T_c cuprates, the Knight shift K^S is proportional to the uniform spin susceptibility $\chi_m(Q=0,\nu=0)$. For example, according to Ref. 4, in $YBa_2Cu_3O_7$

$$\begin{aligned} {}^{63}K^S_{\|} &= \frac{A_{\|}+4B}{{}^{63}\gamma_n\gamma_e\hbar^2}\chi_m(Q=0,\nu=0), \ {}^{63}K^S_{\perp} = \frac{A_{\perp}+4B}{{}^{63}\gamma_n\gamma_e\hbar^2}\chi_m(Q=0,\nu=0), \\ {}^{17}K^S_{\beta} &= \frac{A_{\perp}+4B}{{}^{17}\gamma_n\gamma_e\hbar^2}\chi_m(Q=0,\nu=0), \ {}^{89}K^S = \frac{A_{\perp}+4B}{{}^{89}\gamma_n\gamma_e\hbar^2}\chi_m(Q=0,\nu=0), \end{aligned} \tag{24}$$

${}^{63}K^S_{\|}$, ${}^{63}K^S_{\perp}$, ${}^{17}K^S_{\beta}$, and ${}^{89}K^S$ are all proportional to $\chi_m(Q=0,\nu=0)$, with different ratios determined by the on-site coupling strength and the transferred hyperfine coupling strength of the Cu^{2+} spin to the ${}^{63}Cu$, ${}^{17}O$ and ${}^{89}Y$ nuclei. Here the indices $\|$ and $\perp$ refer to the direction of the static magnetic field. $A_{\|}$, $A_{\perp}$, B, C_{β} and D are hyperfine coupling constants.

As for the ${}^{63}Cu$ nuclear spin echo decay rate $1/({}^{63}T_{2G})$ in the paramagnetic state of high T_c cuprates, Pennington and Slichter [5] showed that

$${}^{63}T_{2G}^{-2} = \frac{0.69}{128\hbar^2}\left[\frac{1}{N}\sum_Q {}^{63}F_{eff}(Q)^2\chi'_m(Q,0)^2 - \left(\frac{1}{N}\sum_Q {}^{63}F_{eff}(Q)\chi'_m(Q,0)\right)^2\right], \tag{25}$$

where $\chi'_m(Q,0)$ is the real part of the dynamical spin susceptibility at momentum Q and frequency 0. ${}^{63}F_{eff}(Q)$ is defined in Ref. 4 as

$${}^{63}F_{eff} = \{A_{\|} + 2B[\cos(Q_x a) + \cos(Q_y a)]\}^2 \tag{26}$$

$$A_{\|} = -4B. \tag{27}$$

We can extract the susceptibility $\chi'_m(Q,\nu=0)$ at real frequency $\nu=0$ from the DCA calculation result on the imaginary frequency axis, using $\chi'_m(Q,\nu=0) = \chi_m(Q,i\nu=0)$.

C. Spectral Function

See Fig. 1 for analytically continued spectral functions.

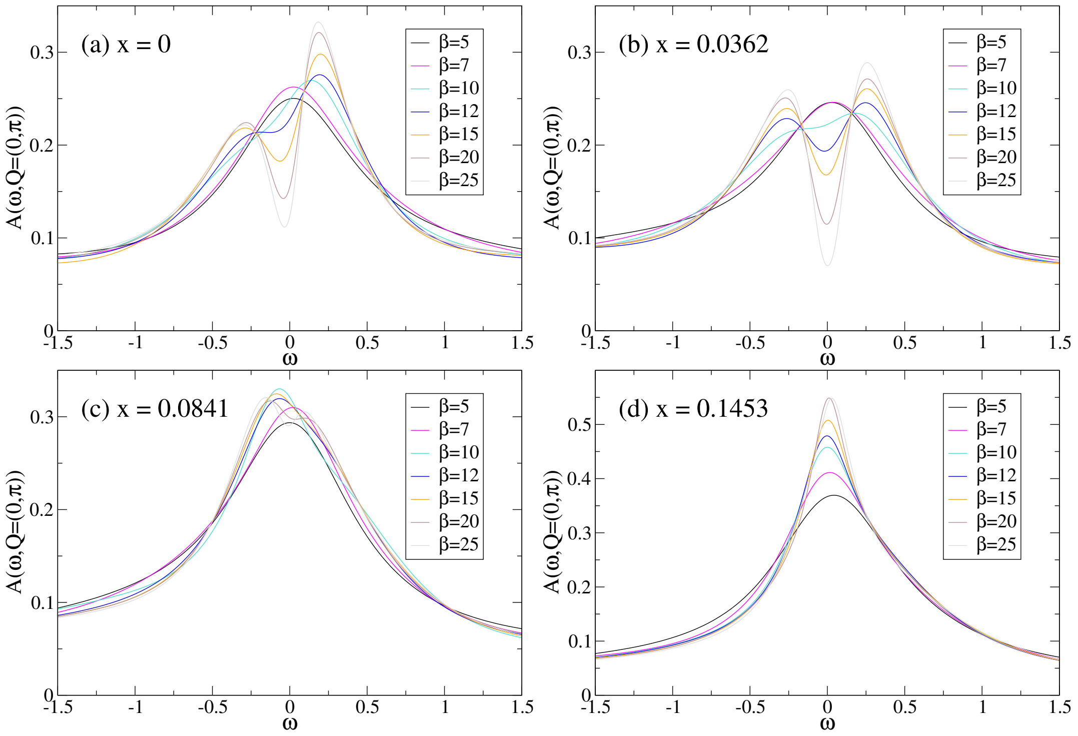

FIG. 1: Spectral function $A(\omega, Q = (0, \pi))$ for 8 site Hubbard model with $U = 6t$, $t' = -0.1t$, $x = 0, 0.0362, 0.0831, 0.1453$, obtained using DCA and Maxent.

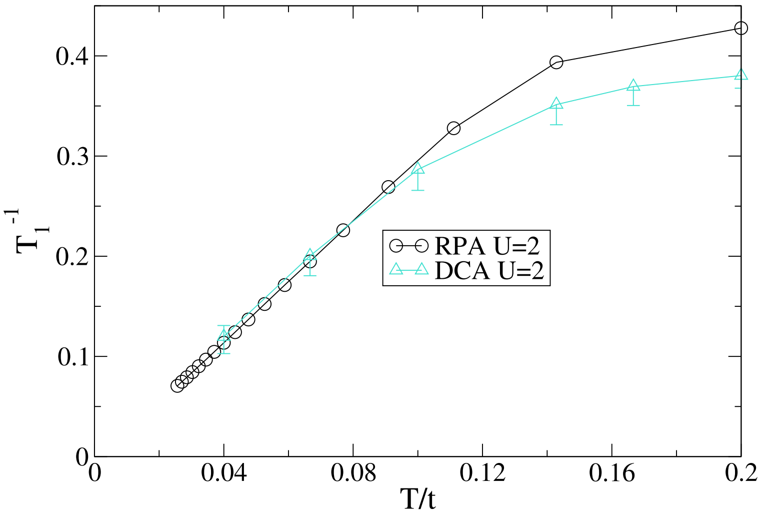

FIG. 2: Spin lattice decay rate of 8-site Hubbard model at $U = 2t$, $t' = 0$. Black solid line, open circle symbol: RPA results. Green solid line with error bar: DCA results.

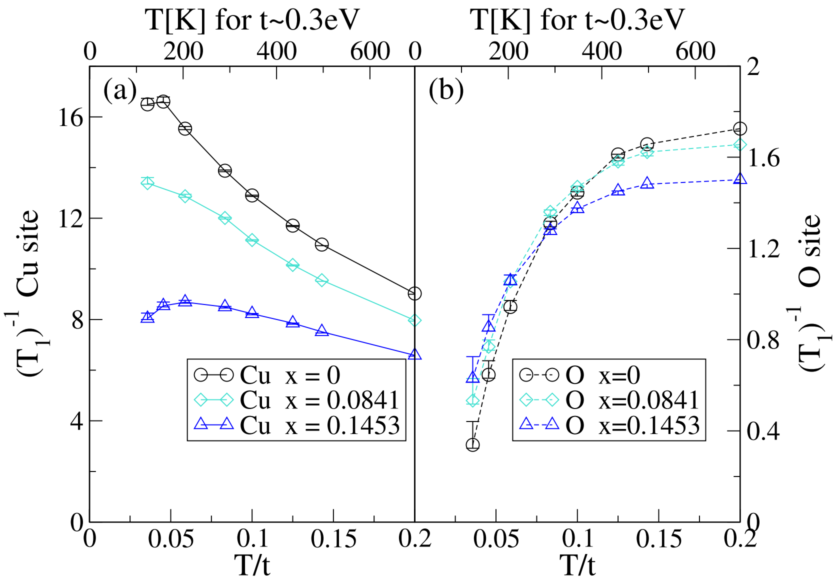

FIG. 3: $(T_1)^{-1}$ plotted as a function of temperature at $U = 6t$, $t' = -0.1t$, for $x = 0$ to $x = 0.145$, by 8-site DCA. Panel (a), solid lines: symmetry factors corresponding to ^{63}Cu site. Panel (b), dashed line: ^{17}O site.

III. SUPPLEMETARY RESULTS

A. Spin Lattice relaxation rate T_1^{-1}

Fig. 2 shows that DCA and RPA calculations agree in the small interaction, low temperature region. Their discrepancy at higher temperature is expected, as explained in Eq. 8 in the main text, as the quality of the approximation of $\frac{1}{T_1}$ becomes better as $T \to 0$.

Fig. 3 shows T- and doping-dependence of $(T_1)^{-1}$ of ^{63}Cu for 3 doping levels. It increases with reduced temperature at 0 doping. Towards larger hole doping, it becomes smaller and evolves to a curve that bends down at low temperature. These features agree with $LSCO_x$ experiment in the low temperature region (Fig. 3 in Ref. 6, Fig. 2 in Ref. 7). At higher temperature $(T_1)^{-1}$ is temperature independent in experiment. The approximation made in Eq. 8 in the main text prevent us from getting accurate $(T_1)^{-1}$ in this regime.

According to Eq. 17, to get T_1^{-1}, one needs to sum bosonic frequency ν from 0 to ∞. However, the numerical expense for obtaining additional frequencies grows $\sim \nu^3$. Although $\chi(i\nu)$ decays rapidly, the maximum frequencies $\frac{2\pi n}{\beta}$ that can be obtained by direct computation are insufficient at low temperature. However, as shown in Fig. 5, the high frequency behavior of $\chi(i\nu)$ is almost temperature independent. Therefore, attaching the high frequency tail to the truncated low temperature curve is a method to extend the region over which it can be summed. We first do a polynomial fitting to the $\beta = 5$ curve, then using this fitted tail as an estimation of high frequency $\chi(i\nu)$ for lower temperatures. The error bars are estimated based on the discrepancy of the fitted polynomial and actual χ_m, which we believe yields the largest contribution to the error, and do not include other sources of error. Note in particular that the numerical error of $\chi(Q, i\nu)$, which is presumably

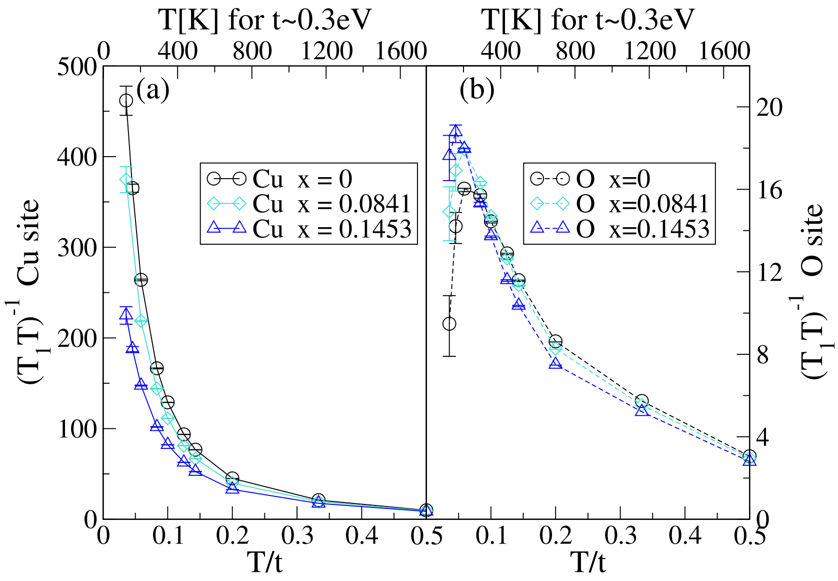

FIG. 4: $(T_1T)^{-1}$ plotted as a function of temperature at $U = 6t$, $t' = -0.1t$, for $x = 0$ to $x = 0.145$ extended to higher temperature, by 8-site DCA. Panel (a), solid lines: symmetry factors corresponding to ^{63}Cu site. Panel (b), dashed line: ^{17}O site.

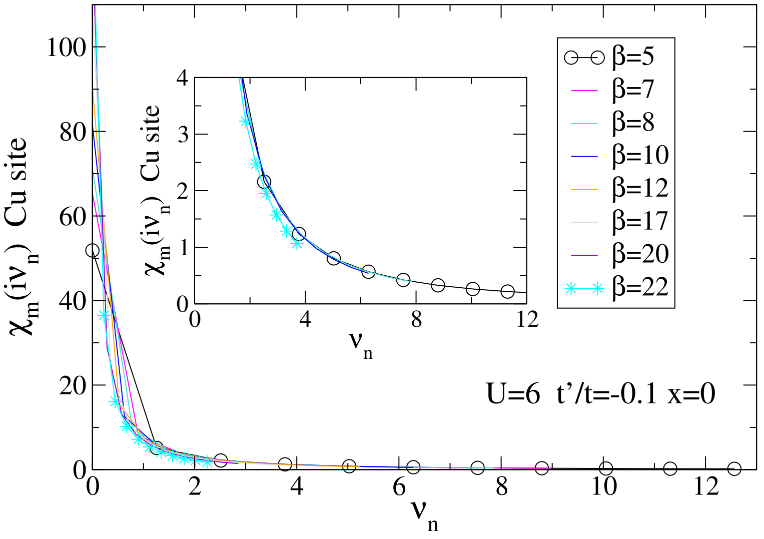

FIG. 5: Spin susceptibility on Matsubara frequency of 8-site Hubbard model at $U = 6t$, $t' = -0.1t$ at different temperature.

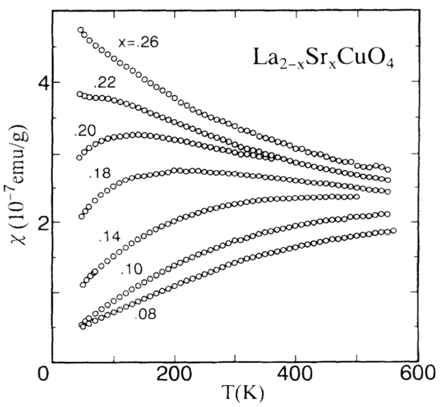

(a) Magnetic susceptibility for the superconducting samples of $La_2Sr_xCuO_4$. (Fig.4 in Ref. 8)

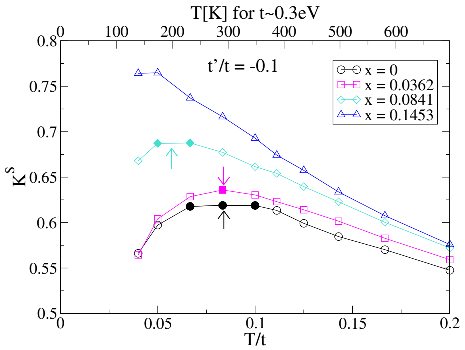

(b) Knight shift $K^S \propto \chi_m$ as a function of temperature T/t (lower x-axis) for a series of doping levels computed at $U = 6t$, $t' = -0.1t$ obtained from 8-site DCA. Filled symbols: the peak positions of the Knight shift. Arrows: onset of normal state pseudogap obtained by analytical continuation of the single particle spectral function at $K = (0, \pi)$. Upper x-axis: T/t in units of Kelvin assuming $t \sim 0.3eV$.

FIG. 6: Side-by-side comparison of Knight shift.

much smaller, is not included.

B. Comparison with experiment

In this final section we present a series of direct comparison figures in order to clarify the relation between data presented in the main text and existing experimental data. Fig. 6 shows a comparison for the Knight shift, Fig. 7 for the relaxation time, and Fig. 8 for the spin-echo decay time.

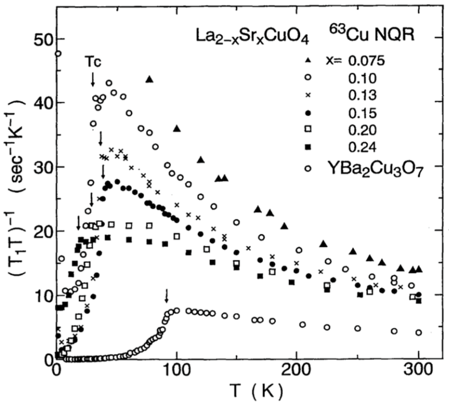

(a) Temperature dependence of $1/(T_1T)$ of Cu for $La_{2-x}Sr_xCuO_4$. $x = 0.075(\blacktriangle)$, $0.1(\circ)$, $0.13(\times)$, $0.15(\bullet)$, $0.2(\square)$, $0.24(\blacksquare)$. Data for $YBa_2Cu_3O_7$ plotted for comparison. (Fig.4 in Ref. 6)

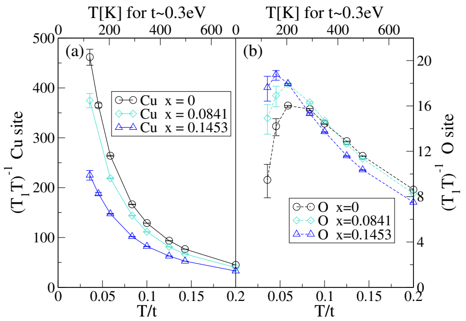

(b) $(T_1T)^{-1}$ plotted as a function of temperature at $U = 6t$, $t' = -0.1t$, for $x = 0$ to $x = 0.145$, by 8-site DCA. Panel (a), solid lines: symmetry factors corresponding to ^{63}Cu site. Panel (b), dashed line: ^{17}O site (See supplemental material for explanation of uncertainties).

FIG. 7: Side-by-side comparison of $(T_1T)^{-1}$ for copper and oxygen sites.

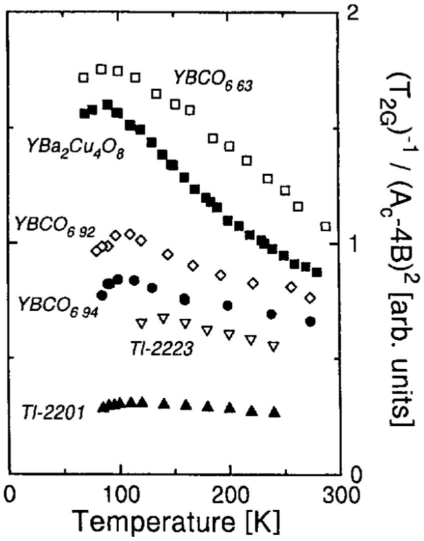

(a) Temperature dependence of $1/T_2$ in various material. (Fig. 8 in Ref. 9)

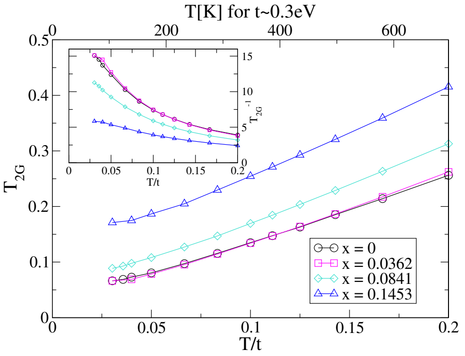

(b) Spin echo decay time T_{2G} as a function of temperature for doping level ranging from $x = 0$ to $x = 0.145$, calculated at $U = 6t$, $t' = -0.1t$. Inset: spin echo decay rate T_{2G}^{-1}.

FIG. 8: Side-by-side comparison of T_{2G} and its inverse.

IV. SUPPLEMENTARY REFERENCES

[1] G. Rohringer, A. Valli, and A. Toschi, Phys. Rev. B **86**, 125114 (2012).
[2] T. Maier, M. Jarrell, T. Pruschke, and M. H. Hettler, Rev. Mod. Phys. **77**, 1027 (2005).
[3] H. Fotso, S. Yang, K. Chen, S. Pathak, J. Moreno, M. Jarrell, K. Mikelsons, E. Khatami, and D. Galanakis, *Dynamical Cluster Approximation* (Springer-Verlag Berlin Heidelberg, 2012).
[4] V. Barzykin and D. Pines, Phys. Rev. B **52**, 13585 (1995).
[5] C. H. Pennington and C. P. Slichter, Phys. Rev. Lett. **66**, 381 (1991).
[6] S. Ohsugi, Y. Kitaoka, K. Ishida, G. qing Zheng, and K. Asayama, Journal of the Physical Society of Japan **63**, 700 (1994).
[7] T. Imai, C. P. Slichter, K. Yoshimura, and K. Kosuge, Phys. Rev. Lett. **70**, 1002 (1993).
[8] T. Nakano, M. Oda, C. Manabe, N. Momono, Y. Miura, and M. Ido, Phys. Rev. B **49**, 16000 (1994).
[9] C. Berthier, M. H. Julien, M. Horvatić, and Y. Berthier, Journal de Physique I **6**, 2205 (1996).
